# Structure-Function Analysis of the HrpB2-HrcU Interaction in the *Xanthomonas citri* Type III Secretion System

**DOI:** 10.1371/journal.pone.0017614

**Published:** 2011-03-09

**Authors:** Paola A. Cappelletti, Rafael Freitas dos Santos, Alexandre M. do Amaral, Rafael Augusto Homem, Thaís dos Santos Souza, Marcos A. Machado, Chuck S. Farah

**Affiliations:** 1 Departamento de Bioquímica, Instituto de Química, Universidade de São Paulo, São Paulo, São Paulo, Brazil; 2 EMBRAPA Recursos Genéticos e Biotecnologia, Brasília, Distrito Federal, Brazil; 3 Centro APTA Citros “Sylvio Moreira”/IAC, Cordeiropolis, São Paulo, Brazil; Instituto Butantan, Brazil

## Abstract

Bacterial type III secretion systems deliver protein virulence factors to host cells. Here we characterize the interaction between HrpB2, a small protein secreted by the *Xanthomonas citri* subsp. citri type III secretion system, and the cytosolic domain of the inner membrane protein HrcU, a paralog of the flagellar protein FlhB. We show that a recombinant fragment corresponding to the C-terminal cytosolic domain of HrcU produced in *E. coli* suffers cleavage within a conserved Asn264-Pro265-Thr266-His267 (NPTH) sequence. A recombinant HrcU cytosolic domain with N264A, P265A, T266A mutations at the cleavage site (HrcU_AAAH_) was not cleaved and interacted with HrpB2. Furthermore, a polypeptide corresponding to the sequence following the NPTH cleavage site also interacted with HrpB2 indicating that the site for interaction is located after the NPTH site. Non-polar deletion mutants of the *hrcU* and *hrpB2* genes resulted in a total loss of pathogenicity in susceptible citrus plants and disease symptoms could be recovered by expression of HrpB2 and HrcU from extrachromossomal plasmids. Complementation of the *ΔhrcU* mutant with HrcU_AAAH_ produced canker lesions similar to those observed when complemented with wild-type HrcU. HrpB2 secretion however, was significantly reduced in the *ΔhrcU* mutant complemented with HrcU_AAAH,_ suggesting that an intact and cleavable NPTH site in HrcU is necessary for total functionally of T3SS in *X. citri* subsp. citri. Complementation of the *ΔhrpB2 X. citri* subsp. citri strain with a series of *hrpB2* gene mutants revealed that the highly conserved HrpB2 C-terminus is essential for T3SS-dependent development of citrus canker symptoms *in planta*.

## Introduction

Many Gram-negative bacterial pathogens produce proteinaceous pathogenic factors that are secreted and injected into the host cell via the type III secretion system (T3SS) during the infective process [1], [2], [3]. A great deal of focus has been aimed at understanding the T3SS of phytopathogenic *Xanthomonas* species that infect a wide variety of plant hosts, many of which are of great economic importance [1], [4], [5], [6], [7], [8], [9], [10], [11], [12], [13], [14], [15]. The phytopathogen *Xanthomonas citri* subsp. citri (*Xanthomonas axonopodis* pv citri strain 306; *Xac*) is the causal agent of citrus canker, a disease that threatens citrus crops world-wide [16]. The *Xac hrp* locus (*hrp*: “hypersensitive response and pathogenicity”) encompasses a group of 25 genes that code for a T3SS. Some products encoded by these genes are conserved in all T3SS, including core flagellar secretory components, while others are proteins of unknown function but whose homologs are essential for T3SS function in other *Xanthomonas* species [5], [7], [17].

The *Xac* T3SS is required for the development of disease symptoms in susceptible citrus plants as well as for the hypersensitive response (HR) in resistant plants [17], [18]. Deletions in the *hrpB* and *hrpD* operons and deletions of the *hrpF* gene in *Xac* failed to produce canker in citrus plants or hypersensitive response (HR) in cotton [17]. Furthermore, a specific T3SS substrate, PthA (a member of the AvrBs3 family), has been shown to contribute significantly to T3SS-dependent development of disease symptoms by *Xac* in citrus and the introduction of the *pthA* gene into strains of *X. phaseoli* and *X. campestris* pv. *malvacearum* (neither pathogenic in citrus) resulted in the elicitation of HR in their respective hosts, bean and cotton [18]. A PthA homolog coded by the *hssB3.0* gene was found to be required for virulence of *Xac* KC21 on *Citrus grandis* cultivars [19]. Other possible T3SS-related factors have been identified in the *Xac* genome by bioinformatics analysis [7] but have not been studied at the genetic or protein level.

We have previously identified protein-protein interactions involving components, substrates and regulators of the T3SS of *Xac* strain 306 [5] whose genome has been sequenced [7]. One of the interactions identified was that involving HrpB2 and HrcU. HrpB2 is a small protein found associated with the T3SS of only a few phytopathogenic bacteria (*Xanthomonas* spp., *Ralstonia solanacearum, Acidovorax avenae*) and of *Burkholderia* spp that can infect animals and plants. In *Xanthomonas campestris* pv. vesicatoria (*Xcv*), HrpB2 is secreted and is essential for the secretion of the AvrBs3 virulence protein by the T3SS [20] and has been shown to interact with HpaC, a protein required for the efficient secretion of other effectors proteins [21]. These observations have led to the suggestion that HrpB2 may play a role in controlling the hierarchy of a stepwise secretion process [20], [21].

HrcU homologs are found in all known T3SSs and flagellar systems and are made up of an N-terminal domain containing several transmembrane helices and a cytoplasmic C-terminal domain. In *Xanthomonas campestris* pv. *glycines* 8ra, the HrcU homolog is not required for HR induction on non-host plants, pepper and tomato, or for the multiplication of bacteria in the host plant, but was required for the pathogenic symptoms on soybean [22]. On the other hand, insertion mutagenesis in the *Xcv hrpC* operon, which codes for both HrcU and HrcV, resulted in nonpathogenic mutants that exhibited significantly reduced growth in pepper leaves and lost the ability to induce HR in resistant host plants and in non-hosts [23].

HrcU is a paralog of the flagellar protein FlhB. The 173-residue C-terminal domain of FlhB from *Salmonella* is specifically cleaved between Asn-269 and Pro-270 within a NPTH motif [24] via an autocatalytic process [25]. This NPTH motif is conserved in all FlhB homologs, including those found in T3SS of animal and plant pathogens and a similar cleavage has been observed in the homolog YscU from the T3SS of *Yersinia pseudotuberculosis* [26]. In flagellar systems, mutations that abolish cleavage in FlhB also abolish the secretion of flagellin and other late export extracellular components but not early export proteins such as FlgD [27]. Cleavage of YscU does not however seem to be essential for the secretion of virulence factors by the *Yersinia* T3SS [26] and thus appears to discriminate between translocator and effector proteins [28]. Substitutions of N263 abolish autocleavage of YscU while P264 and H266 showed partial cleavage [29]. Structural studies of YcsU [29] and its homologs EscU from enteropatogenic *E. coli*, SpaS from *Salmonella typhimurium* [30] and Spa40 from *Shigella flexneri* [31] reported similar structural and functional data.

In this report, we have characterized the interaction between HrpB2 and the C-terminal domain of HrcU of *Xac* using purified recombinant proteins. We show that when expressed in *E. coli*, HrcU_XAC_ suffers a cleavage within the NPTH motif in a manner similar to that observed for the HrcU homologs FlhB and YscU and that the HrpB2_XAC_ binding site on HrcU_XAC_ corresponds to the region C-terminal to the cleavage site. Deletion mutations in the *hrcU* and *hrpB2* genes (Δ*hrcU* and Δ*hrpB2*) resulted in a total loss of virulence *in planta* and pathogenicity could be regained by the expression of HrcU_XAC_ and HrpB2_XAC_ from extrachromosomal plasmids. Furthermore, citrus canker symptoms could be observed in infections of the Δ*hrcU* mutant expressing a HrcU_XAC_ variant in which the NPTH site has was abolished. We also show that HrpB2_XAC_ is secreted in a manner that depends on HrcU_XAC_ but is only partly dependent on HrcU_XAC_ cleavage. Expression of HrpB2_XAC_ variants in a Δ*hrpB2* background showed that the last seven amino acids are essential for HrpB2_XAC_ function in the development of canker disease symptoms.

## Results

### Expression of the cytosolic domain of HrcU_XAC_ (HrcU_XAC_207–357_) in *E. coli* produces a 7 kDa polypeptide

**Figure 1:**
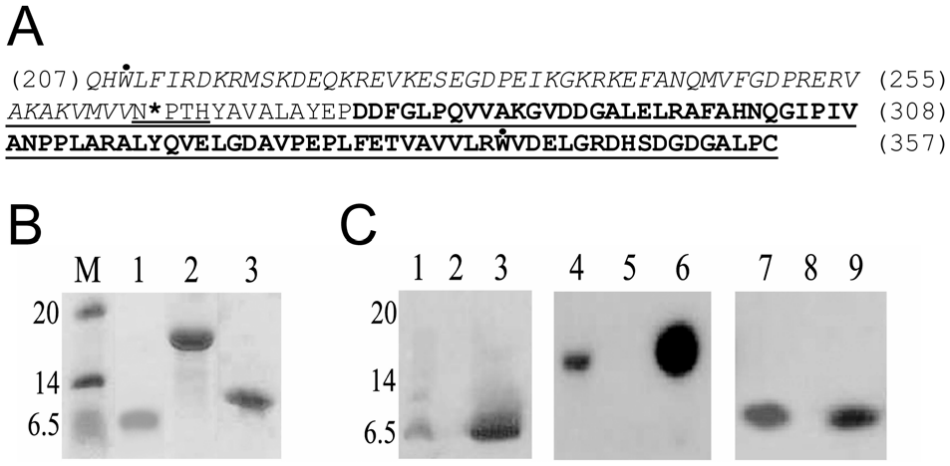
Expression of HrcU_XAC_ C-terminal fragments. **A**) Primary sequence of the C-terminal domain (residues 207-357) of HrcU_XAC_. Residues 207-264 are in *italic* and residues 277-357 are shown in *bold*. The underlined sequence was shown to interact with HrpB2_XAC_ in yeast two-hybrid assays [5]. In HrcU_XAC_207-264_ and HrcU_XAC_207-357(AAAH)_, residues Q207 and H208 were replaced with Met and Asp residues respectively. The highly conserved NPTH sequence is double-underlined and the cleavage site between N264 and P265 is indicated with an asterisk. The two tryptophan (W209 and W340) residues are indicated with a dot above their letter symbols. **B**) Coomassie-stained SDS-PAGE of purified recombinant HrcU fragments. Purified HrcU_XAC_208-264_ (lane 1), HrcU_XAC_207-357AAAH_ (lane 2) and HrcU_XAC_His277-357_ (lane 3). Molecular mass markers (M) are shown on the left with masses in kDa. **C**) Western blots of purified HrcU_XAC_ fragments (lanes 3, 6, 9) and of *E. coli* cell lysates before (lanes 2, 5, 8) and after (lanes 1, 4, 7) expression using the polyclonal antiserum raised against HrcU_XAC_207-357AAAH_. HrcU_XAC_207-357_ (lanes 1-3), HrcU_XAC_207-357AAAH_ (lanes 4-6), HrcU_XAC_His277-357_ (lanes 7-9).

The C-terminal domain of HrcU corresponding to residues 207–357 (HrcU_XAC_207–357_, sequence shown in Fig. 1A) was expressed in *E. coli* BL21(DE3) cells. The expression of the recombinant protein was expected to produce a 158 residue, 17 kDa polypeptide. However, SDS-PAGE analysis failed to detect a 17 kDa fragment but instead a 7 kDa fragment appeared in Coomassie-stained gels after induction with IPTG (data not shown). This fragment was subsequently purified (Fig. 1B, lane 1). This result was obtained after expression in a variety of different *E. coli* strains including BL21(DE3), BL21(DE3)RP, BL21(DE3)RIL, BL21(DE3)pLysS, BL21(DE3)CY, BL21(DE3)SI and BL21(DE3)Star (data not shown).

### Mutation of the conserved NPTH site results in the production of a full-length 17 kDa polypeptide

HrcU orthologs and paralogs all possess a conserved Asn-Pro-Thr-His (NPTH) sequence (residues 264–267 in HrcU_XAC_, Fig. 1A) which has been shown to be a site of auto-cleavage in the flagellar protein FlhB [24], [25]. To test the hypothesis that a similar cleavage was occurring in HrcU_XAC_207–357_, we mutated residues 264–266 to alanine and expressed the polypeptide in *E. coli*. As shown in Figure 1B (lane 2), expression and purification of HrcU_XAC_207–357(AAAH)_ produced a protein of the expected size (17 kDa).

To test whether the 7 kDa fragment was in fact derived from HrcU_XAC_207–357_ we used purified HrcU_XAC_207–357(AAAH)_ to obtain polyclonal antiserum against the HrcU_XAC_ C-terminal domain. Western blot assays against lysates of *E. coli* cultures obtained before and after IPTG-induced expression of HrcU_XAC_207–357_ and HrcU_XAC_207–357(AAAH)_ showed that the antibody recognizes the 17 kDa HrcU_XAC_207–357(AAAH)_ fragment (Fig. 1C, lanes 4 and 5) as well as the 7 kDa fragment (Fig. 1C, lanes 1 and 2). The antibody also recognized the purified 7 kDa fragment (Fig. 1C, lane 3). These results indicate that the purified 7 kDa fragment obtained after HrcU_207–357_ expression is in fact derived from HrcU_XAC_.

N-terminal sequencing by Edman degradation of the 7 kDa fragment was consistent with the N-terminus beginning at position 207 (XXXLFIRDKR), indicating that the initiation Met residue was indeed retained. The mass of the purified 7 kDa fragment determined by MALDI-ToF analysis was very close to the mass expected from the N-terminal fragment (6911 Da with retention of the initiation methionine) produced from cleavage between residues Ans264 and Pro265 of the NPTH sequence within HrcU_XAC_207–357_. The above results thus allow us to designate the name HrcU_XAC_207–264_ to the 7 kDa polypeptide that was detected and purified after expression of HrcU_207–357_.

Cleavage of HrcU_XAC_207–357_ between residues Asn264 and Pro265 would be expected to produce two fragments, one N-terminal fragment beginning at residue 207 and ending at residue 264 (6911 Da) and one C-terminal fragment corresponding to residues 265–357 (9931 Da). As mentioned above, only a 7 kDa fragment could be observed to be induced in Coomassie-stained gels (data not shown). While no 10 kDa fragment was observed to be induced in Coomassie-stained gels, a faint band could be observed above the 7 kDa band in the Western blot of *E. coli* lysates after induction of HrcU_207–357_ expression with IPTG (Fig. 1C, lane 1). Therefore, the evidence so far is consistent with the cleavage at residue 264 possibly followed by a degradation of a significant fraction of the 10 kDa fragment in *E. coli*.

### Interactions between HrpB2_XAC_ and fragments derived from the cytosolic C-terminal domain of HrcU_XAC_

We have previously shown that HrpB2_XAC_ interacts with fragments derived from the C-terminal domain of HrcU_XAC_ in yeast two-hybrid assays [5]. In that study, the smallest HrcU_XAC_ fragment observed to interact corresponded to residues 256 to 357 (underlined sequence in Fig. 1A). It was therefore not clear whether HrcU_XAC_ sequences before or after the conserved NPTH site (or both) were necessary for interaction with HrpB2. We therefore expressed and purified recombinant HrpB2_XAC_ to perform *in vitro* interaction assays with HrcU_XAC_207–264_ and HrcU_XAC_207–357(AAAH)_. We also expressed and purified an HrcU_XAC_ fragment corresponding to residues 277–357 with an N-terminal His-tag fusion (HrcU_XAC_His277–357_) (Figure 1B, lane 3). This fragment is recognized by polyclonal anti-HrcU_XAC_ antibodies both in *E*. *coli* lysates and after purification (Figure 1C, lanes 7, 8 and 9) and its estimated mass determined by MALDI-ToF spectrometry corresponds well with the expected mass of a fragment in which the initiation methionine has been retained (data not shown).

**Figure 2:**
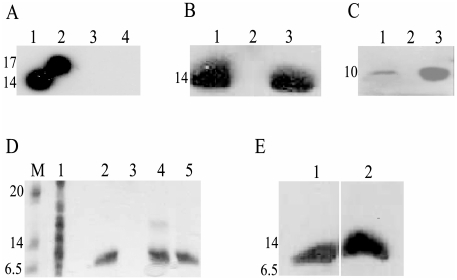
Interaction of HrpB2_XAC_ with HrcU_XAC_207-357AAAH_ and with HrcU_XAC_His277-357_. **A**) Far-Western blot assays demonstrating the HrpB2_XAC_ interaction with immobilized HrcU_XAC_207-357AAAH_. The following purified proteins were separated by SDS-PAGE and transferred to a nitrocellulose membrane: Lane 1: HrpB2_XAC_. Lanes 2 and 4: HrcU_XAC_207-357AAAH._ Lane 3: chicken muscle tropomyosin fragment Tm_143-284_ [60]. Nitrocellulose strips corresponding to lanes 2 and 3 were incubated with HrpB2 followed by washing to remove unbound proteins. Nitrocellulose strips corresponding to lanes 1 to 4 were then incubated with polyclonal antiserum raised against HrpB2_XAC_. The strips were rejoined and revealed using anti-mouse IgG conjugated with horseradish peroxidase. **B**) Far-Western Blot assays demonstrating the HrcU_XAC_207-357AAAH_ interaction with immobilized HrpB2_XAC_. The following samples were separated by SDS-PAGE and transferred to a nitrocellulose membrane: Lysates of *E. coli* cells after (lane 1) and before (lane 2) expression of HrpB2_XAC_ and purified HrpB2_XAC_ (lane 3). The nitrocelulose membrane was incubated with HrcU_XAC_207-357AAAH_ following by incubation with polyclonal antiserum raised against HrcU_XAC_207-357AAAH_ and revealed using protein A conjugated with horseradish peroxidase. **C**) Far-Western Blot assays demonstrating the HrpB2_XAC_ interaction with immobilized HrcU_XAC_His277-357_. The following samples were separated by SDS-PAGE and transferred to a nitrocellulose membrane: Lysates of *E. coli* cells after (lane 1) and before (lane 2) expression of HrcU_XAC_His277-357_ and purified HrcU_XAC_His277-357_ (lane 3). The nitrocellulose membrane was incubated with HrpB2 following by incubation with polyclonal antiserum raised against HrpB2_XAC_ and revealed as described in part A. **D**) Pull-down assay demonstrating the interaction of HrpB2_XAC_ with HrcU_XAC_His277-357_ immobilized on Ni^2+^-chelating resin. HrcU_XAC_His277-357_ (lane 2), HrpB2_XAC_ (lane 3), a HrpB2_XAC_ plus HrcU_XAC_His277-357_ mixture (lane 4) and a mixture of HrcU_XAC_His277-357_ with an *E. coli* BL21(DE3) cell lysate (lane 5) were applied to a Ni^2+^-chelating resin, washed with buffer containing 25 mM imidazole and bound proteins were eluted by washing with 500 mM imidazole. Eluted proteins were separated by SDS-PAGE and visualized by Coomassie brilliant blue staining. Lane 1 shows the contents of the *E. coli* lysate employed in lane 5. Molecular mass markers (M) are shown to the left in kilodaltons. **E**) Since HrpB2_XAC_ and HrcU_XAC_His277-357_ are not easily separated by SDS-PAGE, the presence of both HrpB2_XAC_ and HrcU_XAC_His277-357_ in the bound fraction shown in lane 4 of Figure 2D was demonstrated by Western blot using polyclonal antisera raised against HrcU_XAC_207-357AAAH_ (lane 1) and against HrpB2_XAC_ (lane 2). The masses of molecular weight markers (in kDa) are indicated to the left of parts A-E.

Figure 2A shows the results of Far-Western blot analysis of the interaction between HrpB2_XAC_ and HrcU_XAC_207–357(AAAH)_ using polyclonal antibodies raised against HrpB2_XAC_. HrpB2_XAC_ bound to immobilized HrcU_XAC_207–357(AAAH)_ (lane 2) but not to an immobilized recombinant C-terminal chicken α-tropomyosin fragment used as a negative control (lane 3). Similar experiments using immobilized HrcU_XAC_207–264_ failed to detect an interaction (data not shown). Figure 2B shows that Far-Western assays using immobilized cell lysates obtained before (lane 2) and after induction (lane 1) of HrpB2_XAC_ expression as well as purified HrpB2_XAC_ (lane 3). After incubation of the membranes with HrcU_XAC_207–357(AAAH)_, bound HrcU_XAC_207–357(AAAH)_ could be detected with polyclonal anti-HrcU_XAC_ antibodies. Again, no interactions could be detected in similar experiments in which membranes were incubated with HrcU_XAC_207-264_ (data not shown).

HrcU_XAC_His277–357_ corresponds to a fragment that begins 10 residues after the conserved NPTH site. Binding of HrcU_XAC_His277–357_ to HrpB2_XAC_ was demonstrated in Far-Western experiments using *E. coli* lysates obtained after induction of expression of HrpB2_XAC_ as well as purified HrpB2_XAC_. These samples were submitted to SDS-PAGE, transferred to nitrocellulose membranes, overlayed with HrcU_XAC_His277–357_ and bound HrcU_XAC_His277–357_ was detected using anti-HrcU_XAC_ antibodies (Fig. 2C). This interaction was further demonstrated by immobilizing HrcU_XAC_His277–357_ on a Ni^2+^-chelating resin and testing whether it could retain HrpB2_XAC_ (Fig. 2D and 2E). While purified HrpB2_XAC_ did not interact with the Ni^2+^-chelating resin on its own (Fig. 2D, lane 3), it was retained by HrcU_XAC_His277–357_ bound to the column (Fig. 2D, lane 4 and Fig. 2E, lane 2). Since HrpB2_XAC_ (14 kDa) and HrcU_XAC_His277–357_ (10 kDa) have similar mobility in SDS-PAGE, we detected the individual components of the complex using HrcU_XAC_-specific and HrpB2_XAC_-specific antisera (Fig. 2E, lanes 1 and 2 respectively).

**Figure 3:**
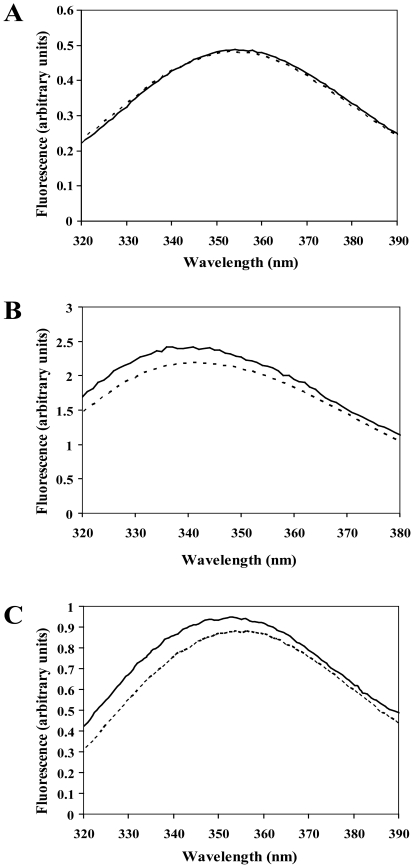
HrpB2_XAC_ induced changes in HrcU_XAC_ fluorescence. Fluorescence emission spectra of HrcU_XAC_207-264_ (A), HrcU_XAC_207-357AAAH_ (B) and HrcU_XAC_His277-357_ (C) in the absence (dotted lines) and presence (solid lines) of HrpB2. All proteins (2 µM) were dissolved in 5 mM sodium acetate (pH 6.0). Spectra were recorded at 25°C using an excitation wavelength of 280 nm.

The specific interaction between HrpB2_XAC_ and the region C-terminal to the HrcU_XAC_ NPTH site was further demonstrated in fluorescence perturbation assays. The HrpB2_XAC_ protein does not possess any tryptophan residues. On the other hand, the C-terminal cytosolic domain of HrcU_XAC_ has two tryptophans, one at position 209, before the NPTH site, and the other at position 340, after the NPTH site (Fig. 1A). We therefore used the intrinsic fluorescence of purified HrcU_XAC_207–357(AAAH)_, HrcU_XAC_207–264_ and HrcU_XAC_His277–357_ as probes to detect interactions with HrpB2. Figure 3 shows that the fluorescence of HrcU_XAC_207–357(AAAH)_ and HrcU_XAC_His277–357_ is perturbed by the addition of HrpB2_XAC_ (Fig. 3B and 3C) while the fluorescence of HrcU_XAC_207–264_ remains unchanged (Fig. 3A). The addition of HrpB2 caused slight blue-shifts in the emission spectra of both HrcU_XAC_207–357(AAAH)_ and HrcU_XAC_His277–357_ as well as a small increase in intensity. These results confirm that the site of HrpB2_XAC_ interaction on HrcU_XAC_ corresponds to the sequence C-terminal to the NPTH cleavage site.

### The NPTH cleavage site is not required for the development of canker symptoms

To study the contribution of HrcU_XAC_ and its NPTH site to *Xac* pathogenicity we employed an allelic exchange protocol to the produce the Δ*hrcU Xac* strain containing in-frame deletions of *hrcU* codons 14-347 (Table 1). We also produced plasmids containing the *hrcU* open reading frame plus 1 kb upstream sequences that contain the promoter region (pUFR047_*hrcU*; Table 2 *)*. Furthermore, we introduced mutations in this plasmid that change the NPTH site to AAAH (pUFR047_*hrcU_AAAH_*).

**Table 1:** Strains used in this study.

*Strains*	*Relevant characteristics*	*Source*
Bacterial Strains:		
*E. coli* DH10B	Recipient for cloning experiments	[56]
*E. coli* BL21(DE3)	IPTG-inducible T7 RNA polymerase	[58]
*E. coli* BL21(DE3) (RIL)	IPTG-inducible T7 RNA polymerase	[59]
*Xac* strain 306	Template for PCR-based cloning	[7]
*Xac* Δ*hrcU*	Xac strain carrying deletion of *hrcU* gene (codons 14–347)	This study
*Xac* Δ*hrpB2*	Xac strain carrying deletion of *hrpB2* gene (codons 10–119)	This study
*Xac* Δ*hrcU*+pUFR047 _*hrcU*	*Xac* Δ*hrcU* carrying pUFR047_*hrcU*	This study
*Xac* Δ*hrcU*+pUFR047_*hrcU_AAAH_*	*Xac* Δ*hrcU* carrying pUFR047_*hrcU_AAAH_*	This study
*Xac* Δ*hrpB2*+pUFR047_*hrpB2*	*Xac* Δ*hrpB2* carrying pUFR047_*hrpB2*	This study
*Xac* Δ*hrpB2*+pUFR047_*hrpB2_1-56_*	*Xac* Δ*hrpB2* carrying pUFR047_*hrpB2_1-56_*	This study
*Xac* Δ*hrpB2*+pUFR047_*hrpB2_1-123_*	*Xac* Δ*hrpB2* carrying pUFR047_*hrpB2_1-123_*	This study
*Xac* Δ*hrpB2*+pUFR047_*hrpB2_LQGPR_*	*Xac* Δ*hrpB2* carrying pUFR047_*hrpB2_LQGPR_*	This study
*Xac* Δ*hrpB2*+pUFR047_*hrpB2_T125A_*	*Xac* Δ*hrpB2* carrying pUFR047_*hrpB2_T125A_*	This study
*Xac* Δ*hrpB2*+pUFR047_*hrpB2_L126A_*	*Xac* Δ*hrpB2* carrying pUFR047_*hrpB2_L126A_*	This study
*Xac* Δ*hrpB2*+pUFR047_*hrpB2_V127A_*	*Xac* Δ*hrpB2* carrying pUFR047_*hrpB2_V127A_*	This study
*Xac* Δ*hrpB2*+pUFR047_*hrpB2_K128A_*	*Xac* Δ*hrpB2* carrying pUFR047_*hrpB2_K128A_*	This study
*Xac* Δ*hrpB2*+pUFR047_*hrpB2_N129A_*	*Xac* Δ*hrpB2* carrying pUFR047_*hrpB2_N129A_*	This study
*Xac* Δ*hrpB2*+pUFR047_*hrpB2_Q130A_*	*Xac* Δ*hrpB2* carrying pUFR047_*hrpB2_Q130A_*	This study

*Strains*	*Relevant characteristics*	*Source*
Bacterial Strains:		
*E. coli* DH10B	Recipient for cloning experiments	[56]
*E. coli* BL21(DE3)	IPTG-inducible T7 RNA polymerase	[58]
*E. coli* BL21(DE3) (RIL)	IPTG-inducible T7 RNA polymerase	[59]
*Xac* strain 306	Template for PCR-based cloning	[7]
*Xac* Δ*hrcU*	Xac strain carrying deletion of *hrcU* gene (codons 14–347)	This study
*Xac* Δ*hrpB2*	Xac strain carrying deletion of *hrpB2* gene (codons 10–119)	This study
*Xac* Δ*hrcU*+pUFR047 _*hrcU*	*Xac* Δ*hrcU* carrying pUFR047_*hrcU*	This study
*Xac* Δ*hrcU*+pUFR047_*hrcU_AAAH_*	*Xac* Δ*hrcU* carrying pUFR047_*hrcU_AAAH_*	This study
*Xac* Δ*hrpB2*+pUFR047_*hrpB2*	*Xac* Δ*hrpB2* carrying pUFR047_*hrpB2*	This study
*Xac* Δ*hrpB2*+pUFR047_*hrpB2_1-56_*	*Xac* Δ*hrpB2* carrying pUFR047_*hrpB2_1-56_*	This study
*Xac* Δ*hrpB2*+pUFR047_*hrpB2_1-123_*	*Xac* Δ*hrpB2* carrying pUFR047_*hrpB2*_1-123_	This study
*Xac* Δ*hrpB2*+pUFR047_*hrpB2_LQGPR_*	*Xac* Δ*hrpB2* carrying pUFR047_*hrpB2*_LQGPR_	This study
*Xac* Δ*hrpB2*+pUFR047_*hrpB2_T125A_*	*Xac* Δ*hrpB2* carrying pUFR047_*hrpB2*_T125A_	This study
*Xac* Δ*hrpB2*+pUFR047_*hrpB2_L126A_*	*Xac* Δ*hrpB2* carrying pUFR047_*hrpB2*_L126A_	This study
*Xac* Δ*hrpB2*+pUFR047_*hrpB2_V127A_*	*Xac* Δ*hrpB2* carrying pUFR047_*hrpB2*_V127A_	This study
*Xac* Δ*hrpB2*+pUFR047_*hrpB2_K128A_*	*Xac* Δ*hrpB2* carrying pUFR047_*hrpB2*_K128A_	This study

*See Table 2 for plasmid construction details.

**Figure 4:**
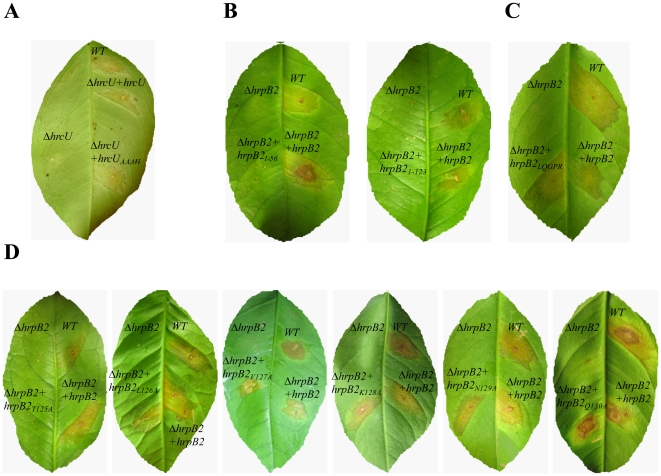
HrcU and HrpB2 contribute to *Xac* pathogenicity during infection of *Citrus sinensis*. Macroscopic symptoms 15 days after inoculation on the abaxial surface of leafs with Δ*hrcU* (**A**) and Δ*hrpB2* (**B-D**) mutants. The following strains were used: *Xac* wild-type (WT), Δ*hrcU*, Δ*hrcU*+pUFR047_*hrcU* (Δ*hrcU*+*hrcU*), Δ*hrcU*+pUFR047_*hrcU_AAAH_* (Δ*hrcU*+*hrcU_AAAH_*), Δ*hrpB2,* Δ*hrpB2+*pUFR047*_hrpB2* (Δ*hrpB2+hrpB2)*, Δ*hrpB2+*pUFR047*_hrpB2_1-56_* (Δ*hrpB2+hrpB2_1-56_*), Δ*hrpB2+*pUFR047*_hrpB2_1-123_* (Δ*hrpB2+hrpB2_1-123_*), Δ*hrpB2+pUFR047_hrpB2_LQGPR_* (Δ*hrpB2+hrpB2_LQGPR_)*, Δ*hrpB2+*pUFR047*_hrpB2_T125A_* (Δ*hrpB2+hrpB2_T125A_*), Δ*hrpB2+*pUFR047*_hrpB2_L126A_* (Δ*hrpB2+hrpB2_L126A_*), Δ*hrpB2+*pUFR047*_hrpB2_V127A_* (Δ*hrpB2+hrpB2_V127A_*), Δ*hrpB2+*pUFR047*_hrpB2_K128A_* (Δ*hrpB2+hrpB2_K128A_*), Δ*hrpB2+*pUFR047*_hrpB2_N129A_* (Δ*hrpB2+hrpB2_N129A_*) and Δ*hrpB2+*pUFR047*_hrpB2_L130A_* (Δ*hrpB2+hrpB2_L130A_*).

Figure 4A shows the results of inoculation of sweet orange leaf tissue with the *Xac* wild-type, Δ*hrcU, ΔhrcU+*pUFR047*_hrcU and ΔhrcU+*pUFR047_*hrcU_AAAH_* strains 15 days after infection. While infection with the wild-type strain showed clear disease symptoms including water-soaking, hyperplasy and necrosis, the Δ*hrcU* strain failed to produce any disease symptoms in the susceptible citrus host. This result is consistent with the absolute requirement for HrcU homologs for the functioning of all T3SS systems [22], [32]. The phenotype of the Δ*hrcU* strain could be reverted by the expression of wild-type HrcU coded by the pUFR047_*hrcU* plasmid or by expression of the HrcU_XAC_AAAH_ coded by the pUFR047_*hrcU_AAAH_* plasmid (Fig. 4A). In both cases, canker symptoms were less severe than those observed using the wild-type strain. It is not clear why the reversion of disease symptoms was attenuated in these experiments. We note that the native upstream promoter regions contained within these plasmids contain PIP (plant-inducible promoter) boxes [7] that have been shown to be recognized by the HrpX transcription factor that controls *hrp* expression in *Xcv* [33], [34], [35].

### The HrpB2_XAC_ C-terminal region is required to elicit citrus canker symptoms

To study the contribution of HrpB2_XAC_ to *Xac* pathogenicity, the allelic exchange protocol was used to produce the Δ*hrpB2* strain with an in-frame deletion of *hrpB2* codons 10-119 (Table 1). We also produced plasmid pUFR047_*hrpB2* (Table 2) which codes for the wild-type HrpB2_XAC_ protein plus a 1 kb upstream region that includes the *hrpB1* gene between the promoter and *hrpB2*. Figure 4B shows that the Δ*hrpB2* strain was unable to elicit disease symptoms and that the virulence of the mutant strain was fully restored by transformation with pUFR047_*hrpB2*.

**Table 2:** Plasmids used in this study.

*Plasmids*	*Relevant characteristics*	*Source*
pET-11d	T7 RNA polymerase - based expression vector	[57]
pET-3a	T7 RNA polymerase - based expression vector	[57]
pET-28a (+)	T7 RNA polymerase - based expression vector	Novagen
pU1	pET-11d based vector expressing HrcU_XAC_207-357_	This study
pU2	pET-11d based vector expressing HrcU_XAC_207-357(AAAH)_	This study
pU3	pET-28a(+) based vector expressing HrcU_XAC_His277-357_	This study
pB2	pET-3a based vector expressing HrpB2_XAC_	This study
pET-Tmy_143-284_	pET-3a based vector expressing chicken alpha tropomyosin	[60]
pNPTS138	Suicide vector, Km^r^/*SacB*	Dickon Alley*
pUFR047	Wide host range vector, Gm^r^	[63]
pBBR1MCS-5	Wide host range vector, Gm^r^	[62]
pNPTS138_Δ*hrcU*	Suicide vector carrying internal truncation of *Xac hrcU* gene	This study
pNPTS138_Δ*hrpB2*	Suicide vector carrying internal truncation of *Xac hrpB2* gene	This study
pBBR_*hrcU*	pBBR1MCS-5 vector carrying *Xac hrcU* gene	This study
pBBR_*hrcU_AAAH_*	pBBR1MCS-5 vector carrying *Xac hrcU* gene with mutations that change NPTH motif to AAAH	This study
pUFR047_*hrcU*	pUFR047 based vector for expression of HrcU_XAC_ in *Xac*	This study
pUFR047_*hrcU_AAAH_*	pUFR047 based vector for expression of HrcU_XAC_AAAH_ in *Xac*	This study
pBBR_*hrpB2*	pBBR1MCS-5 vector carrying *Xac hrpB2* gene	This study
pUFR047_*hrpB2*	pUFR047 based vector for expression of HrpB2_XAC_ in *Xac*	This study
pUFR047_*hrpB2_1_*_-56_	pUFR047 based vector for expression of HrpB2_XAC_1-56_ in *Xac*	This study
pUFR047_*hrpB2_1_*_-123_	pUFR047 based vector for expression of HrpB2_XAC_1-123_ in *Xac*	This study
pUFR047_*hrpB2_LQGPR_*	pUFR047 based vector for expression of HrpB2_XAC_LQGPR_ in *Xac*	This study
pUFR047_*hrpB2_T125A_*	pUFR047 based vector for expression of HrpB2_XAC_T125A_ in *Xac*	This study
pUFR047_*hrpB2_L126A_*	pUFR047 based vector for expression of HrpB2_XAC_L126A_ in *Xac*	This study
pUFR047_*hrpB2_V127A_*	pUFR047 based vector for expression of HrpB2_XAC_V127A_ in *Xac*	This study
pUFR047_*hrpB2_K128A_*	pUFR047 based vector for expression of HrpB2_XAC_K128A_ in *Xac*	This study
pUFR047_*hrpB2_N129A_*	pUFR047 based vector for expression of HrpB2_XAC_N129A_ in *Xac*	This study
pUFR047_*hrpB2_Q306A_*	pUFR047 based vector for expression of HrpB2_XAC_Q130A_ in *Xac*	This study

*unpublished.

**Figure 5:**
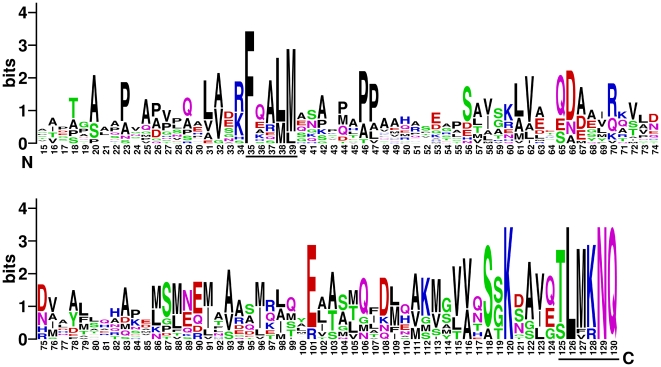
Graphical Representation of the multiple sequence alignment of the HrpB2 protein family. The Pfam database [64] lists 61 sequences in this group (PF09487) from *Xanthomonas* (13 sequences), *Burkholderia* (43 sequences), *Ralstonia* (3 sequences), and *Acidovorax* (2 sequences) species. However, after removal of all sequences with greater that 95% identity, only 16 remain. These 16 sequences were used to generate this representation using the WebLogo server (http://weblogo.berkeley.edu/) [65] in which the height of the residue symbol indicates the degree of conservation (the representation obtained using all 61 sequences is highly similar). Numbers refer to residue positions in HrpB2_XAC_. The FQALM and TLMKNQ motifs are underlined.

Multiple sequence alignment analysis of HrpB2 proteins from *Xanthomonas*, *Burkholderia*, *Acidovorax* and *Ralstonia* species (Figure 5) indicates that there are two regions of sequence conservation in an otherwise variable protein family: i) a five residue motif which we name FQALM that corresponds to positions 35–39 of HrpB2_XAC_ and ii) the last six amino acids of the protein (HrpB2_XAC_ residues 125–130) which we name the TLMKNQ motif (in *Xac* the methionine residue is substituted with a valine).

In order to determine whether either or both of these motifs is important for HrpB2_XAC_ function in the elicitation of citrus canker symptoms, we expressed HrpB2_XAC_ fragments or full-length HrpB2_XAC_ variants (Table 1) in the Δ*hrpB2* strain. To test the importance of the FQALM motif we mutated these residues to LQGPR and expressed the mutant protein (HrpB2_XAC_LQGPR_) in the Δ*hrpB2* strain using the pUFR047*_hrpB2_LQGPR_* plasmid. The Δ*hrpB2+*pUFR047*_hrpB2_LQGPR_* strain was able to cause citrus canker symptoms in a manner indistinguishable from the wild-type *Xac* strain (Fig. 4B). Therefore, the FQALM motif does not seem to be essential for HrpB2 function. When the Δ*hrpB2* strain was transformed with plasmids pUFR047*_hrpB2_1-56_* and pUFR047*_hrpB2_1-123_*, leading to the expression of HrpB2_XAC_1-56_ and HrpB2_XAC_1-123_ respectively, neither of the resulting strains were able to induce citrus canker symptoms in orange leaves (Fig. 4C). These results suggested that the C-terminal region of HrpB2_XAC_ which contains the conserved TLMKNQ motif is important for HrpB2 function. To test the importance of each residue in this motif, six *hrpB2*_XAC_ mutants in which each of these residues were changed to alanine were expressed the Δ*hrpB2* strain. The results showed while the *Xac* strain expressing HrpB2_XAC_T125A_ was not able produce canker symptoms, the strains expressing HrpB2_XAC_V127A_, HrpB2_XAC_K128A_, HrpB2_XAC_N129A_ and HrpB2_XAC_Q130A_ produced canker symptoms to the same extent as wild-type *Xac*. Furthermore, Δ*hrpB2* cells expressing H*rpB2_XAC_L126A_* produced attenuated citrus canker symptoms when compared to the same cells containing the plasmid that expresses wild-type HrpB2_XAC_ (Fig. 4D). These results point to the importance of the TLMKNQ motif, and especially to the first residue of this motif (T125), in the role of HrpB2_XAC_ in the development disease in citrus plants.

### HrpB2_XAC_ is secreted by *Xac* in liquid media

**Figure 6:**
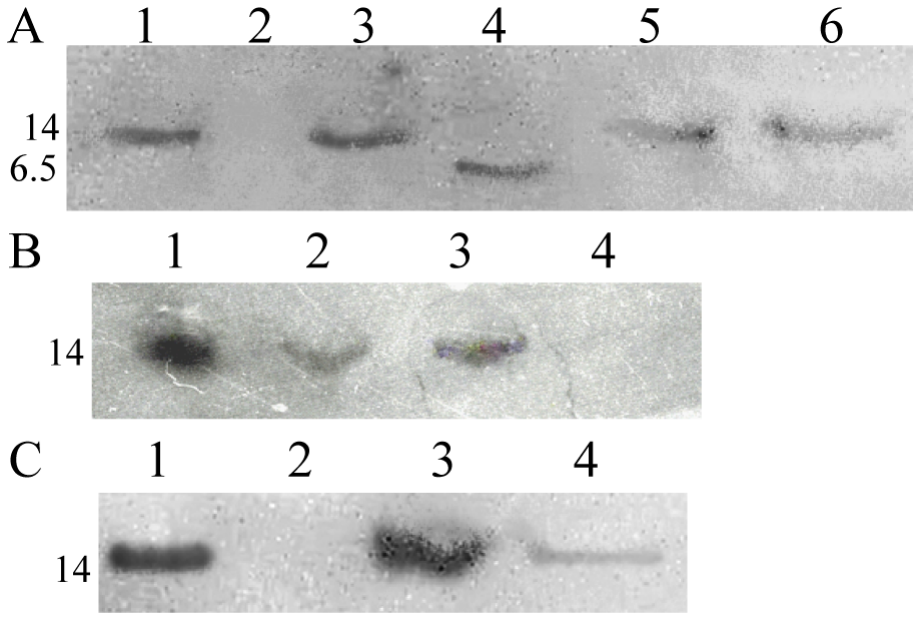
HrpB2_XAC_ is secreted by *Xac*. Liquid cultures of *Xac* were grown as described in Materials and Methods. Secreted fractions were concentrated and separated by SDS-PAGE 18% and proteins were transferred to nitrocellulose membranes. HrpB2_XAC_ was detected using anti-HrpB2_XAC_ antiserum and revealed using anti-mouse IgG conjugated with horseradish peroxidase. (**A**) Lane 1: *Xac* wild-type, lane 2: *Xac* Δ*hrpB2,* lane 3: *Xac* Δ*hrpB2+*pUFR047_*hrpB2*, lane 4: *Xac ΔhrpB2+*pUFR047*_hrpB2_1-56_*, lane 5: *Xac* Δ*hrpB2+*pUFR047*_hrpB2_1-123_*, lane 6: *Xac* Δ*hrpB2+*pUFR047*_hrpB2_LQGPR_*. (**B**) Lane 1: *Xac* wild-type, lane 2: *Xac ΔhrpB2+*pUFR047*_hrpB2_T125A_*, lane 3: *Xac* Δ*hrpB2+*pUFR047*_hrpB2_Q130A_,* lane 4: *Xac* Δ*hrpB2*. (**C**) Lane 1: *Xac* wild-type, lane 2: *Xac* Δ*hrcU*, lane 3: *Xac* Δ*hrcU*+pUFR047_*hrcU* and lane 4: *Xac* Δ*hrcU*+pUFR047_*hrcU_AAAH_*.

Rossier *et al.* [20], showed that in *Xcv* HrpB2 is secreted in a T3SS-dependent manner. In that study, a mutant *Xcv* strain with constitutive expression of the *hrp* locus (due to a constitutively activated HrpG mutation) was used. No such mutant *Xac* strain has yet been isolated or produced. Expression of *hrp* genes in *Xcv* is dependent on unknown plant signals and is controlled by specific promoters with PIP boxes [33], [34], [35]. In *Ralstonia solanacearum, hrp* expression is dependent on contact with an unidentified component derived from the host cell wall [36], [37], [38] and passion fruit leaf extracts have been shown to modify the proteome of *X. axonopodis* pv. passiflorae [39]. We therefore grew liquid *Xac* cultures in the presence of extracts derived from sweet orange (*C. sinensis*) leaves. Proteins in the secreted fraction were separated by SDS-PAGE and probed for HrpB2_XAC_ by Western blot analysis using anti-HrpB2_XAC_ antiserum. We found that HrpB2_XAC_ could be observed in the secreted fraction of wild-type cells (Fig. 6A, lane 1). As expected, secretion of HrpB2_XAC_ was abolished in the Δ*hrpB2* mutants and complementation with pUFR047_*hrpB2* restored HrpB2_XAC_ secretion (Fig. 6A, lanes 2 and 3, respectively). We did not detect HrpB2_XAC_ in the cellular fractions (data not shown) but note that the secreted fraction was concentrated 60-fold in relation to the cellular fraction (see Experimental Procedures).

We then asked whether the HrpB2_XAC_ mutants described above were secreted when expressed in the Δ*hrpB2* strain. Figure 6A (lanes 4, 5 and 6) shows that HrpB2_1-56_ (5.7 kDa), HrpB2_1-123_ (13 kDa) and HrpB2_XAC_LQGPR_ were all observed in *Xac* culture supernatants. Furthermore, all six mutants carrying alanines at each position of the TLMKNQ motif could be detected in *Xac* culture supernatants (Fig. 6B, lanes 2 and 3 and data not shown). Finally, we observed that HrpB2_XAC_ secretion was abolished in the Δ*hrcU* mutant (Fig. 6C, lane 2). Complementation of the Δ*hrcU* mutant with pUFR047_*hrcU* restored HrpB2_XAC_ secretion to wild-type levels (Fig. 6C, lane 3). Interestingly, complementation of the Δ*hrcU* mutant with pUFR047_*hrcU_AAAH_* resulted in significantly reduced levels of HrpB2_XAC_ secretion (Fig. 6, lane 4). This difference in levels of HrpB2_XAC_ secretion may, therefore, be due to the inability of the HrcU_XAC_AAAH_ protein to undergo the self-cleavage reaction. Apparently, only minimal amounts of HrpB2_XAC_ are necessary to elicit citrus canker symptoms during the infection process.

### HrcU_XAC_ is not required for *Xac* survival *in planta*

**Figure 7:**
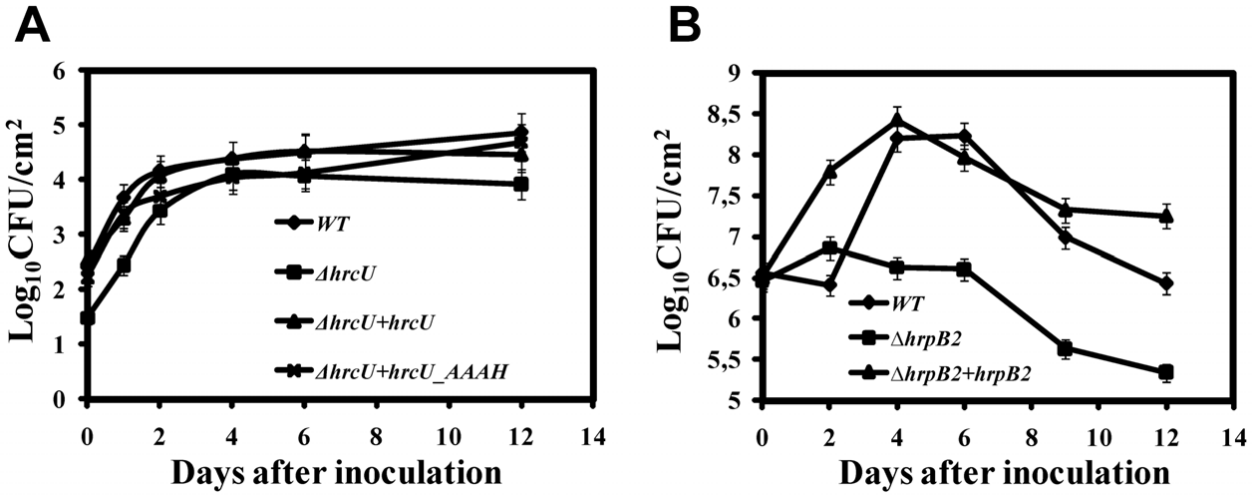
Number of colony-forming units (CFU) of *Xac* strains per cm^2^ of leaf tissue during the first twelve days after inoculation. The abaxial surface of young leaves was pricked by using insect pins whose tips were previously immersed in the bacterial suspension for *Xac hrcU* mutant strains (A) or by infiltration into leaves with needleless syringes for *Xac hrpB2* mutant strains (B). Discs of infected leaves were excised, homogenized and cultured quantitatively by incubation on agar plates. The assays were performed in triplicate and error bars represent the standard deviation of the data. Differences in the initial bacterial populations are due to differences in the inoculation protocols. (A) *Xac* wild-type (diamonds), Δ*hrcU* (squares), *ΔhrcU*+pUFR047_*hrcU* (triangles), Δ*hrcU*+pUFR047_*hrcU_AAAH_* (crosses). (B) *Xac* wild-type (diamonds), Δ*hrpB2* (squares), *ΔhrpB2*+pUFR047_*hrpB2* (triangles).

In order to determine whether HrcU_XAC_ and its NPTH site were necessary for *Xac* survival in inoculated host leafs, we inoculated Citrus leafs with *Xac* bacterial suspensions and accompanied bacterial numbers during a 12 day period after infection. Figure 7A shows that wild-type *Xac*, Δ*hrcU*, Δ*hrcU*+pUFR047_*hrcU*, Δ*hrcU*+pUFR047_*hrcU_AAAH_* strains all presented similar growth curves. This suggests that HrcU_XAC_ is not absolutely required for bacterial survival *in planta*, in spite of the fact that the Δ*hrcU* does not produce canker disease symptoms. In contrast, the Δ*hrpB2* strain presented significantly reduced survival when compared to the wild-type and Δ*hrpB2*+pUFR047_*hrpB2* strains (Fig. 7B).

## Discussion

In this study we constructed non-polar knock-out mutants for the *hrcU* and *hrpB2* genes and show that they completely abolish pathogenicity of *Xac* in sweet orange. Complementation of the Δ*hrcU* strain with plasmids pUFR047_*hrcU* or pUFR047_*hrcU_AAAH_* recovered the capacity to induce disease symptoms. We also demonstrated that HrpB2_XAC_ is secreted to the extracellular space in a HrcU_XAC_-dependent manner by the *Xac* T3SS. HrpB2_XAC_ secretion was abolished in the Δ*hrcU* knockout and restored in the Δ*hrcU*+pUFR047_*hrcU* and Δ*hrcU*+pUFR047_*hrcU_AAAH_* strains, but the amount of HrpB2_XAC_ secreted by the Δ*hrcU*+pUFR047_*hrcU_AAAH_* strain was reduced with respect to that observed for the wild-type and Δ*hrcU*+pUFR047_*hrcU* strains (Figure 6C). This result suggests that while HrcU_XAC_ cleavage may not be absolutely necessary for the proper functioning of the *Xac* T3SS, it may contribute to the efficiency by which it carries out its tasks.

In this report we have shown that HrcU_XAC_ expressed in *E. coli* suffers proteolysis at a highly conserved NPTH site in a manner similar to that already described for its paralog FlhB of the flagellar system [24] and its orthologs YscU, EscU and SpaS from the T3SSs of *Yersina* [26], [40], *E. coli* and *Salmonella* [30] respectively. This, and a similar report for the HrcU protein from *Xanthomonas campestris* pv. *vesicatoria* [21], are the first observations of NPTH-dependent cleavage of a FlhB homolog from the T3SS of a plant pathogen. Ferris *et al.* [25] have shown that FlhB cleavage at the NPTH site is an autocatalytic process; that is, FlhB catalyzes its own hydrolysis at this site. Furthermore, a series of crystal structures of the C-terminal domains of the FlhB homologs EscU and SpaS from the *E. coli* and *Salmonella* T3SSs [30], and YscU from *Yersinia enterocolitica* [29] have recently provided information regarding the mechanism and conformational changes associated with self-cleavage.

We also show that the HrcU_XAC_ C-terminal fragment that is released upon HrcU_XAC_ self-cleavage interacts with HrpB2_XAC_, whose only known homologs are found in the phytopathogens *Xanthomonas* spp., *Ralstonia solanacearum*, and *Acidovorax avenae*, as well as *Burkholderia* spp that infect both animals and plants. Our results show that HrpB2_XAC_ does not interact specifically with the site of HrcU_XAC_ cleavage since it could bind to HrcU_XAC_207-357(AAAH)_ and to HrcU_His277-357_, a fragment that begins 10 residues after the NPTH site. Unfortunately we were not able to detect HrcU_XAC_ in the wild-type or complemented mutant strains using the anti-HrcU_XAC_ polyclonal antibodies in this study (data not shown) and so could not determine relative levels of HrcU_XAC_ in the *Xac* strains nor have we so far been able to determine whether HrcU_XAC_ is in fact cleaved at the NPTH site in *Xac* cells. However, during the preparation of this work, HrcU cleavage was observed in *Xcv* [21].

In order to understand HrcU function it is useful to recall what we know about the functioning of HrcU homologs. Mutants that inhibit cleavage at the NPTH site of HrcU homologs exhibit defects in the secretion of specific substrates. For example, in FlhB, mutations at this site inhibit the export of “late” flagellar proteins, while normal levels of early substrates, including hook protein FlgE, are secreted [27]. Also, a *Y. enterocolitica* Δ*yscU* strain expressing YscU_N263A_, in which the conserved Asn residue of the NPTH sequence was mutated to Ala, produced longer needles, exported reduced amounts of YscP (a FliK homolog, see below) and did not export the translocator proteins LcrV, YopB and YopD. The first two defects could be compensated by overexpression of YscP (see below) while export of LcrV, YopB and YopD was absolutely dependent on a cleavable NPTH site [28].

The cleaved FlhB C-terminal fragment binds to both early and late flagellar export substrates (FlgD, FliC). Furthermore, the product of the *fliK* gene, FliK or flagellar hook-length control protein, binds to the self-cleavage C-terminal fragment of FlhB [24] and during flagellar assembly FliK is itself secreted subsequent to hook protein subunit secretion [41], [42]. Also, *fliK* mutants do not secrete late substrates but do secrete excessive amounts of hook protein (FlgE), resulting in the production of characteristic polyhooks [43]. This phenotype can be reverted by single amino acid substitutions in FlhB, almost all of which map to the C-terminal self-cleavage fragment [27], [43], [44], [45]. Thus, in the flagellar system, FlhB and FliK act together to control substrate switching from early to late substrates, though the molecular mechanism by which this is achieved is not fully understood [41], [46].

In the animal pathogens *Yersinia*, *Salmonella* and *Shigella*, the formation of needle complexes and subsequent secretion of virulence factors by T3SSs are controlled by an interplay between FlhB and FliK homologs. In these systems, mutations in the FliK homologs YscP [47], [48], InvJ [49] or Spa32 [50] result in the formation of needles of variable length and compromised virulence factor secretion. In the case of *Yersinia*, the phenotypes can be reverted by mutations in the cytosolic domains of the FlhB homolog YscU [47]. Futhermore, YscP, InvJ and Spa32 are secreted during T3SS assembly [50], [51], [52] in a manner similar to the secretion of FliK in the flagellar system. Finally, in *Yersinia*, YscP secretion appears to be coupled to the secretion of another small protein (YscO) that binds preferentially to the uncleaved form of YscU [53].

Few YscP homologs from non-flagellar T3SS have been identified in plant-associated bacteria: HrpP from *Pseudomonas syringae,* RspP from *P. fluorescens,* HpaP from *R. solanacearum* [54] and the HpaP/HpaC proteins coded by the *hrp* gene clusters of *Xanthomonas spp* (for example HpaC in *Xcv* and HpaP in *Xac*). During the preparation of this work Lorenz *et al.* [21] published a study on the HpaC and HrpB2 proteins from *Xcv*. They found that: 1) amino acids 10 to 25 of HrpB2 are crucial for its efficient secretion and function and that HrpB2 is necessary for the secretion of effectors and of extracellular components of the secretion apparatus, 2) HrpB2 and HpaC interact with each other and both also interact with the C-terminal domain of HrcU and 3) HrpB2 secretion is suppressed by HpaC. They therefore speculated that HpaC acts to control the switch between the secretion of early to late T3SS substrates (see also reference [20]) and that HpaC binding to HrcU specifically inhibits HrpB2 binding and secretion [21]. While HpaC from *Xcv* has been shown to be necessary for the secretion of both T3SS effector and translocon proteins, it is not required for the export of the Hrp pilus protein HrpE [55]. In this sense, the *hpaC* mutant phenotype in *Xcv* is similar to that observed for *yscP*, *invJ* and *spa32* mutants (see above). However, HpaC itself is not secreted by *Xcv* and Hrp pilus formation was not affected in *hpaC* mutant strains (different from that observed for *yscP*, *invJ* and *spa32* mutants as described above) [55]. On the other hand, HrpB2 binds to HrcU and, like FliK and YscP, HrpB2 is itself secreted. Since both HrpB2 and HpaC bind to the C-terminal domain of HrcU, the accumulated evidence so far is not clear as to which (if either) HpaC-HrcU or HrpB2-HrcU complexes carry out molecular functions in the *Xanthomonas* T3SS that are orthologous to those of YscU-YscP and FlhB-FliK described above.

One interesting observation from our study was that while both Δ*hrcU* and Δ*hrpB2* knockout strains do not induce citrus canker symptoms, only the latter presents a significant reduction in survival in the host tissue. The Δ*hrcU* mutant survives as well as the wild type strain in the host tissue, but does not detectably secrete HrpB2. A similar phenomena has been observed in *X. campestris* pv. *glycines* 8ra where HrcU is required for pathogenicity in its natural soybean host but is not required for multiplication in the host plant nor is it required for the induction of HR in non-hosts [22]. The molecular basis for the differences in the Δ*hrcU* and Δ*hrpB2* phenotypes in *Xac* is not yet clear. One possibility is that the ΔhrcU mutant fails to secrete effector(s) that trigger specific host defense mechanisms resulting in the bacterial survival. Another possibility is that intracellular HrpB2 may contribute to *Xac* survival while extracelular HrpB2 contributes to citrus canker symptom development.

In *Xcv*, deletion of HrpB2 residues 10-25 impaired protein secretion and disease symptom formation, which led to the conclusion that secretion is required for function [21]. We demonstrated that while Δ*hrpB2*+pUFR047_*hrpB2_1-56_*, Δ*hrpB2*+ pUFR047_*hrpB2_1-123_* and Δ*hrpB2*+pUFR047_*hrpB2_T125A_* strains are not able to cause citrus canker, the truncated HrpB2_XAC_ polypeptides and HrpB2_XAC_ single amino acid substitution mutants are all however secreted to the extracellular space. Therefore, HrpB2_XAC_ secretion, *per se*, is not sufficient for HrpB2_XAC_ function. Apparently, the conserved C-terminal region of the protein, more specifically residue T125 in the conserved TLMKNQ motif, is especially important for HrpB2_XAC_-dependent pathogenicity.

Important unanswered questions remain regarding HrpB2 function at the molecular level. Further studies are needed to determine whether HrpB2 exercises a role in substrate switching or as a minor structural component of the T3SS pilus (as do hook-filament junction and capping proteins in bacterial flagella) or carries out other, as yet not contemplated, functions and also whether these functions are effected within the bacterial cell or in the exterior subsequent to its secretion (or both).

## Materials and Methods

### Construction vectors for the expression of HrcU_XAC_207-357_, HrcU_XAC_His277-357_, HrcU_XAC_207-357(AAAH)_ and HrpB2_XAC_ in *E. coli*

*E. coli* strains and plasmids are described in Table 1 and Table 2, respectively. *E. coli* cells were cultivated at 37°C in 2xYT media [56]. When necessary, the appropriate antibiotics were added at the following final concentrations: ampicillin 200 µg/ml, kanamycin 50 µg/ml and chloramphenicol 200 µg/ml. Synthetic oligonucleotide primers (Table 3) for polymerase chain reactions (PCR) were designed containing restriction sites useful for cloning (see below). PCR products were purified from agarose gels using the QIAquick Gel Extraction Kit (Qiagen). To produce a vector for the expression of HrcU_XAC_207-357_, the DNA sequence coding residues 207-357 of the *hrcU* gene was amplified from genomic *Xac* DNA using the oligonucleotides F-U_207-357_ and R-U_207-357_ (Table 3). The PCR product was digested with endonucleases NcoI and HindIII and inserted into the expression vector pET-11d [57], previously digested with the same enzymes to produce plasmid pU1. Primers F-U_AAAH_ and R-U_AAAH_ (Table 3) were used in PCR with pU1 as template in order to change the codons for residues 264-266 to alanine codons using the QuikChange Site-Directed Mutageneis Kit (Stratagene). The resulting recombinant plasmid (pU2) directs the expression of HrcU_XAC_207-357(AAAH)_. Note that in both recombinant HrcU_XAC_207-357_ (through which HrcU_XAC_207-264_ is purified, see below) and HrcU_XAC_207-357(AAAH)_, residues Gln207 and His208 have been mutated to Met and Asp residues, respectively, due to the introduction of restriction sites used in the cloning protocol. To produce a vector for the expression of HrcU_XAC_His277-357_, the sequence coding for HrcU residues 277-357 was amplified using primers F-U_His277-357_ and R-U_His277-357_ (Table 3). This product was digested with NdeI and HindIII and ligated into the expression vector pET-28a (Novagen) previously digested with the same enzymes to produce the recombinant plasmid pU3. To produce a vector for the expression of full-length HrpB2_XAC_, the expression vector pET-3a (Studier *et al.,* 1990) was digested with HindIII, filled in with the Klenow fragment of *E. coli* DNA polymerase I and then digested with NdeI. Primers F-B2 and R-B2 were used in a PCR with *Xac* genomic DNA, the product was treated with Klenow fragment and polynucleotide kinase to produce blunt ends, digested with NdeI and then ligated into the pET-3a vector described above to produce the recombinant plasmid pB2. The accession numbers for the complete Xac genome sequence and the HrpB2_XAC_ and HrcU_XAC_ protein sequences are NC_003919, NP_640763 and NP_640761, respectively.

**Table 3:** Oligonucleotides used in this study.

*Oligonucleotides*	*Sequence*
F-U_207-357_	5′ CATCCCATGGACTGGCTGTTCATCCGGGAC 3′
R-U_207-357_	5′ CCCAAGCTTCTCGAGGCTCGCACGCGATCTCCTAG 3′
F-U_AAAH_	5′ GTGATGGTGGTCGCCGCGGCCCATTACGCGGTGGCAC
R-U_AAAH_	5′ GTGCCACCGCGTAATGGGCCGCGGCGACCACCATCAC 3′
F-U_His277-357_	5′ TAAATTGCTCATATGGATGACTTCGGCCTA 3′
R-U_His277-357_	5′ TAAATTGCTCCATGGATGACTTCGGCCTA 3′
F-B2	5′ CGGAATTCCATATGACGCTCATTCCTCCTGTC 3′
R-B2	5′ CCGCTCGAGCTATTGGTTCTTGACCAGTGTCTG 3′
F1-U	5′ TCGGGACTAAAGCTTGCATCAACT TGATCT 3′
R1-U	5′ GGAATTACCATATGCAGTTTCTTCTCGGTCGGCTTCTC 3′
F2-U	5′ GGAATTACCATATGCACAGCGACGGCGATGGAGCT 3′
R2-U	5′ TTTGAACTTGCTAGCTGATCGGTGCCGCTG 3′
R-compU	5′ ATTTTAAGCTTGTCGACCTAGCATGGCAGAGCTCC 3′
F1-B2	5′ CACTACAAGCTTAAGCAACCAGCAAGGGGA 3′
R1-B2	5′ GGAATTACCATATGAATCGCTTGGACAGGAGGAAT 3′
F2-B2	5′ GGAATTACCATATGAAGAACGCCGTGCAGACACTG 3′
R2-B2	5′ AACATTAAATCTAGAGTCGACTGGTTCGCATGCAGGCCGAGC 3′
R-compB2	5′ AATTTAAGCTTGTCGACCTATTGGTTCTTGACCAGTGTC3′
F-M57	5′GCAGCGAGTGGGCAACCCGAGCTAGATGAGCCGCGTGGTCGATGTGC3′
R-M57	5′GCACATCGACCACGCGGCTCATCTAGCTCGGGTTGCCCACTCGCTGC3′
F-Q124	5′GCAATCGGGAAAGAACGCAGTGTAGACACTGGTCAAGAATCAATAG3′
R-Q124	5′CTATTGATTCTTGACCAGTGTCTACACTGCGTTCTTTCCCGATTGC3′
F-FQALM	5′CGCTAGTGAATCGCTTACAAGGGCCGAGGCAGTCCTCTAGC3′
R-FQALM	5′GCTAGAGGACTGCCTCGGCCCTTGTAAGCGATTCACTAGCG3′
F-T125A	5′GGAAAGAACGCAGTGCAGGCACTGGTCAAGAATCAATAG3′
R-T125A	5′CTATTGATTCTTGACCAGTGCCTGCACTGCGTTCTTTCC3′
F-L126A	5′GAAAGAATGCAGTGCAGACAGCGGTCAAGAACCAATAGGT3′
R-L126A	5′ACCTATTGGTTCTTGACCGCTGTCTGCACTGCATTCTTTC3′
F-V127A	5′AGAACGCAGTGCAGACACTGGCCAAGAATCAATAGGTCGAC3′
R-V127A	5′GTCGACCTATTGATTCTTGGCCAGTGTCTGCACTGCGTTCT3′
F-K128A	5′GCCGTGCAGACACTGGTAGCAAACCAATAGGTCGACCTCGA3′
R-K128A	5′TCGAGGTCGACCTATTGGTTTGCTACCAGTGTCTGCACGGC3′
F-N129A	5′CCGTGCAGACACTAGTCAAGCGCCAATAGGTCGACCTCGAGGG3′
R-N129A	5′CCCTCGAGGTCGACCTATTGGCGCTTGACTAGTGTCTGCACGG3′
F-Q130A	5′TGCAGACACTGGTCAAGAACGCATAGGTCGACCTCGAGGGGGG3′
R-Q130A	5′CCCCCCTCGAGGTCGACCTATGCGTTCTTGACCAGTGTCTGCA3′

### Expression and purification of recombinant HrpB2_XAC_ and HrcU_XAC_ fragments

Plasmid constructs pU1, pU2, and pB2 were used to transform *E. coli* strain BL21(DE3) [58] and pU3 was used to transform BL21(DE3)RIL cells [59]. The synthesis of recombinant proteins was induced by the addition of 0.4 mM isopropyl-β-D-thiogalactopyranoside when cultures grown at 37°C attained an optical density of 0.8 at 600 nm. After three more hours of growth, cells were collected by centrifugation at 4500 x *g* for 15 min at 4°C and ressuspended in 20 ml/l of culture of 25 mM Tris-HCl (pH 8.0) for HrcU_XAC_ fragments, and 5 mM sodium acetate (pH 6.0) for HrpB2_XAC_. Cells were lysed by passage through a French pressure cell followed by centrifugation at 37000 x *g* for 1 hour at 4°C. Expression of HrcU_XAC_207-357_ led to the production of a 7 kDa polypeptide, not 17 kDa expected from the size of the protein coded by the gene fragment in the pU1 vector (see Results). This polypeptide was purified from the soluble fraction of the bacterial lysate by Q-Sepharose (Amersham Bioscience) anion-exchange chromatography (25 mM Tris-HCl (pH 8.0), 14 mM β-mercaptoethanol) using a 0-300 mM NaCl gradient, followed by Superdex G-75 (Amersham Bioscience) size exclusion chromatography (25 mM Tris-HCl (pH 8.0), 100 mM NaCl, 14 mM β-mercaptoethanol). HrcU_XAC_207-357(AAAH)_ and HrpB2_XAC_ recombinant proteins were recovered from the insoluble fraction of the bacterial lysate by solubilizing in 25 mM Tris-HCl (pH 8.0), 14 mM β-mercaptoethanol, 8 M urea for HrcU_XAC_207-357(AAAH)_ or 5 mM sodium acetate (pH 6.0), 14 mM β-mercaptoethanol, 8 M urea for HrpB2_XAC_. HrcU_XAC_207-357(AAAH)_ was purified by Q-Sepharose anion-exchange chromatography, using the solubilization buffer (above) and a 0-300 mM NaCl gradient followed by Superdex G-75 size exclusion chromatography using 25 mM Tris-HCl (pH 8.0), 100 mM NaCl, 14 mM β-mercaptoethanol, 8 M urea. HrpB2_XAC_ was purified by passing the protein mixture through a Q-sepharose column equilibrated with 5 mM sodium acetate (pH 6.0), 14 mM β-mercaptoethanol, 8 M urea. HrpB2_XAC_ does not bind to this column under these conditions. The unbound fraction containing HrpB2_XAC_ was concentrated using an 10 kDa Amicon filter (Millipore) and separated by Superdex G-75 size exclusion chromatography using 5 mM sodium acetate (pH 6.0), 100 mM NaCl, 14 mM β-mercaptoethanol, 8 M urea. HrcU_XAC_207-357(AAAH)_ and HrpB2_XAC_ were refolded by dialyses against 25 mM Tris-HCl (pH 8.0), 14 mM β-mercaptoethanol for HrcU_XAC_207-357(AAAH)_, or 5 mM sodium acetate (pH 6.0), 14 mM β-mercaptoethanol for HrpB2_XAC_ containing successively reduced amounts of urea: 6 M, 4 M, 2 M, 0 M. HrcU_His277-357_ was purified from the insoluble fraction of the bacterial lysate by solubilizing in 25 mM Tris-HCl (pH 8.0), 10 mM imidazole, 100 mM NaCl, 2 mM β-mercaptoethanol, 8 M urea. The protein mixture was applied to a Ni^2+^-chelating Sepharose column equilibrated with the same buffer and eluted using a 25-500 mM imidazole gradient. HrcU_XAC_His277-357_ fractions were pooled and the protein was refolded by successive dialyses against 25 mM Tris-HCl (pH 8.0), 14 mM β-mercaptoethanol containing 6 M, 4 M, 2 M and 0 M urea.

### Production of polyclonal antibodies against HrcU_XAC207-357(AAAH)_ and HrpB2_XAC_ proteins

Swiss Webster mice were immunized with four injections, separated by one week intervals, of 10 µg soluble HrpB2_XAC_. New Zealand white rabbits were immunized with HrcU_XAC_207-357(AAAH)_ using four 200 µg injections separated by one week intervals. In both cases, the antigens were diluted with one volume of complete Freund's adjuvant (Sigma) for the first immunization and one volume of incomplete Freund's adjuvant (Sigma) for the remaining immunizations. Blood was collected and incubated for 1 hr at 37°C and the serum was recovered by centrifugation at 5000 x g for 15 min at room temperature, aliquoted and stored at -20°C. Before use, antiserum aliquots were incubated with an *E. coli* lysate as described [56].

### Edman degradation N-terminal sequencing

The N-terminus of the 7 kDa polypeptide purified after the expression of HrcU_XAC_207-357_ was lyophilized and dissolved in ultrapure water. N-terminal sequencing was carried out by Edman degradation using a PPSQ/23 sequencer (Shimadzu Corporation, Tokyo).

### Mass spectrometry experiments

Purified proteins were analyzed by Matrix Assisted Laser Desorption Ionization (MALDI) Time of Flight (TOF) Mass Spectrometry (MS) using an Ettan MALDI-TOF Pro system (Amersham Biosciences). All MALDI-TOF MS spectra were externally calibrated using a cytochrome C standard (12327 Da). Protein mass was identified in linear mode with positive ionization at 20 kV. The samples were mixed with an equal volume of sinapinic acid matrix dissolved in 50% acetonitrile, 0.5% of trifluoroacetic acid. A 0.5 µl aliquot was loaded onto stainless steel MALDI slides for analysis. Spectra were analyzed using the Ettan Maldi-Tof Pro v2.0 software package.

### Western blot assays

Samples were separated by SDS-PAGE (18% acrylamide) and electroblotted onto a nitrocellulose membrane. The membrane was colored with Ponceau red to identify the positions of specific proteins and then blocked for 2 h with 10 mM Tris-HCl (pH 7.5), 150 mM NaCl, 0.1% Tween 20, 0.1% Triton (TBS-TT) and 5% non-fat dry milk. The membranes were probed for 2 h with the appropriate polyclonal antiserum in 5-10 ml of the above blocking buffer (1∶3000 dilution for anti-HrcU_XAC_ antibody and 1∶20000 dilution for the anti-HrpB2_XAC_ antibody) and then washed four times for 15 min with TBS-TT. The anti-HrpB2_XAC_ antibody was detected using an anti-mouse IgG conjugated with horseradish peroxidase (Sigma) at a dilution of 1∶6000. The anti-HrcU_XAC_ antibody was detected using protein A conjugated with horseradish peroxidase (Sigma) at a dilution of 1∶30000. The membranes were incubated for 2 h with the protein-A or anti-IgG conjugates in 5-10 ml of blocking buffer following by washing with TBS-TT. Reactive bands were detected using the ECL AdvanceTM Western Blotting Detection Kit (GE Heathcare-Amersham) according to the manufacturer's instructions.

### Far-Western assays

Far-Western blot assays were carried out to detect specific protein-protein interactions. Approximately 15 µg of purified recombinant protein or lysates from *E. coli* cells was separated by SDS-PAGE (18% acrylamide) and electroblotted onto nitrocellulose membranes. The membrane was blocked for 2 h with TBS-TT plus 5% nonfat dry milk followed by 14 h incubation with 50 µg/ml of a second purified recombinant protein (indicated in the figure legends) at 4°C. Unbound proteins were removed by washing the membranes four times for 15 min with TBS-TT. Bound proteins were then detected as described for the Western blot assays (above). In some cases, negative control experiments were performed using a polypeptide derived from residues 143-284 of chicken muscle α-tropomyosin [60].

### His-tag pulldown assays

HrcU_XAC_His277-357_, HrpB2_XAC_ and an *E. coli* lysate were dialyzed at 4°C against 25 mM Tris-HCl, 100 mM NaCl, 2 mM β-mercaptoethanol, 10 mM imidazole (pH 8.0). A mixture of HrcU_His277-357_ (30 µM) and HrpB2 (30 µM) was added to a 0.25-ml aliquot of Ni^2+^-chelating Sepharose resin (Amersham Bioscience) equilibrated in the above buffer at room temperature. In control experiments, the resin was mixed with only HrcU_XAC_His277-357_ or HrpB2 or with a mixture of HrcU_His277-357_ and a lysate derived from 10 ml of *E. coli* BL21(DE3) culture (OD_600_ = 0.8). The mixtures were washed four times with 1 ml of 25 mM Tris-HCl, 100 mM NaCl, 2 mM 2-β-mercaptoethanol, 25 mM imidazole (pH 8.0). Bound proteins were released by washing with 50 µl of 25 mM Tris-HCl, 100 mM NaCl, 2 mM 2-mercaptoethanol, 500 mM imidazole (pH 8.0). Samples were then analyzed by SDS-PAGE and Western blot assay.

### Fluorescence experiments

HrcU_XAC_207-357(AAAH)_ and HrpB2_XAC_ (both 2 µM) were dissolved in 5 mM sodium acetate pH 6.0 at 25°C. Fluorescence emission spectra were obtained using an AVIV (Lakewood, NJ) ATF 105 Automated Titrating Differential/Ratio spectrofluorometer and were collected between 320 and 400 nm using an excitation wavelength of 280 nm and excitation and emission bandwidths of 2 nm and 5 nm respectively.

### Production of *Xac* genes knockouts

Deletion strains were constructed using the suicide vector pNPTS138 (Alley Dickon, unpublished) by allelic exchange as described [61]. DNA fragments (1 kb) flanking each side of the *Xac hrpB2* and *hrcU* genes were amplified by PCR using oligonucleotides listed in Table 3. For *hrcU*, primer pairs F1-U + R1-U and F2-U + R2-U were used. For *hrpB2,* primer pairs F1-B2 + R1-B2 and F2-B2 + R2-B2 were used. The products were digested with endonuclease NdeI and specific pairs were joined together with T4 DNA ligase (New England Biolabs). The resulting fragments were cloned into pNPTS138 generating pNPTS138-Δ*hrcU* using HindIII and NheI and pNPTS138-Δ*hrpB2* by using HindIII and SalI. These plasmids were introduced by electroporation into *Xac* strain 306. Kanamycin and ampicillin-resistant colonies were selected and grown on plates containing 5% sucrose and ampicillin. Sucrose-sensitive and kanamycin- and ampicilin-resistant colonies were selected and used to inoculate 10 ml of 2xYT-ampicilin medium, which was incubated overnight with agitation at 28°C. A 100 µl aliquot of this culture was plated without dilution on 2xYT agar plates containing 200mg/L ampicillin. The resulting colonies were transferred in replica on two plates: one containing kanamycin and ampicillin and another containing sucrose and ampicilin. Clones that were simultaneously kanamycin-sensitive and sucrose-resistant were selected, and the deletion was confirmed by PCR.

### Production of expression vectors for complementation of Δ*hrcU* and Δ*hrpB2 in Xac*

A fragment containing the *hrcU* gene plus 1 kb upstream sequences was amplified by PCR using primers F1-U and R-compU (Table 3). This fragment contains the complete HrcU open reading frame as well as its native promoter. After digestion with HindIII and SalI, this fragment was cloned into the HindIII-SalI sites of pBBR1MCS-5 [62], resulting in pBBR_*hrcU* (Table 2). To construct pBBR_*hrcU_AAAH_* (Table 2), primers F-U_AAAH_ and R-U_AAAH_ (see Table 3) were used in a PCR amplification with pBBR_*hrcU* as template to change the codons for residues 264-266 (NPT) to alanine codons using the QuikChange Site-Directed Mutagenesis Kit (Stratagene). The mutation was confirmed by sequencing. The HindIII/SalI fragments of pBBR_*hrcU* and pBBR_*hrcU_AAAH_*, which contain the complete HrcU_XAC_ open reading frame as well as its native promoter, were cloned into the same sites of pUFR047, a broad-host range vector carrying a gentamycin resistance gene [63], generating constructs pUFR047_*hrcU* and pUFR047_*hrcU_AAAH_* (Table 2). These plasmids were used to transform the Δ*hrcU* mutant strain by electroporation followed by selection on LB plates with 10 µg/ml gentamycin and 200 µg/ml ampicillin.

A fragment containing the *hrpB2* gene plus 1 kb upstream sequences was amplified by PCR using primers F1-B2 and R-compB2 (Table 3), digested with HindIII and cloned into the HindIII site of pUFR047. The resulting construct, pUFR047_*hrpB2* (Table 2) was used to transform the *Xac* Δ*hrpB2* mutant strain by electroporation. Transformed colonies were selected on LB/gentamycin/ampicilin plates to produce strain Δ*hrpB2*+pUFR047*_hrpB2* (Table 1). To produce *hrpB2* gene mutants for expression in *Xac*, the HindIII/SalI fragment of the PCR product above was cloned between the HindIII and SalI sites of pBBR1MCS-5 generating the construct pBBR_*hrpB2* (Table 2) which was then used as a template to produce mutants using the QuikChange Site-Directed Mutagenesis Kit (Stratagene). Primers F-M57 and R-M57, F-Q124 and R-Q124 (Table 3) were used to change the codons 171 and 372 to stop codons; primers F-FQALM and R-FQALM (Table 3) were used to change the codons for the FQALM motif (residues 35-39) to LQGPR codons and finally, primers pairs F-T125A and R-T125A, F-L126A and R-L126A, F-V127A and R-V127A, F-K128A and R-K128A, F-N129A and R-N129A and F-Q130A and R-Q130A (Table 3) were used to change the respective codons to alanine codons. The HindIII/SalI fragments from all these pBBR_*hrpB2* derived constructs were cloned between the same sites of pUFR047 generating the constructions pUFR047*_hrpB2_1-56_*, pUFR047*_hrpB2_1-123_,* pUFR047*_hrpB2_T125A_*, pUFR047*_hrpB2_L126A_*, pUFR047*_hrpB2_V127A_,* pUFR047*_hrpB2_K128A_,* pUFR047*_hrpB2_N129A,_* and pUFR047*_hrpB2_Q130A_* (Table 2). All the mutations were confirmed by sequencing. Theses constructions were used to transform the *Xac* Δ*hrpB2* strain by electroporation (Table 1).

### Plant bioassays

Highly susceptible Navel sweet orange (*Citrus sinensis* (L.) Osbeck) plants were grown under greenhouse conditions and maintained at 28°C with daylight for virulence assays. To visually monitor the development of citrus canker symptoms, *Xac* 306 and mutant strains were grown overnight at 30°C and adjusted to an optical density of 0.3 at 600 nm in 2xYT culture medium. The suspensions were hand-infiltrated with a 1-ml syringe with needle into the abaxial surface of attached leaves. To monitor bacterial growth *in planta*, *Xac* strains were grown overnight at 30°C and adjusted to an optical density at 600 nm (*OD*_600_) of 0.5 in NB culture medium (8g of nutrient broth liter^-1^, 5 g of NaCl liter^−1^, pH 7). The abaxial surface of young leaves was pricked by using pins whose tips were previously immersed in the bacterial suspension for *Xac hrcU* mutant strains (Fig. 7A) or by infiltration into leaves with needleless syringes for *Xac hrpB2* mutant strains (Fig. 7B). In both cases, leaf disks (0.8 cm^2^) from infected plants were removed with a cork borer during a 12 day period post-inoculation, macerated in 0.85% NaCl with a mortar and pestle. Different dilutions were spread on LB plates with the appropriate antibiotics and the bacterial population was determined by counting colonies after a 2-day incubation period at 28-30°C. Experiments were performed in triplicate.

### Preparation of orange leaf extracts

Sweet orange leaf extracts were prepared as described previously for passion fruit leaf extracts [39]. Leaves were washed extensively with sterile water. Midribs were excluded and 1 g of tissue was mixed with liquid nitrogen and pulverized to form a fine powder. One-hundred milliliters of MM medium [39] plus carbenicillin 100 µg/ml, pH 7.4 were added and the mixture was macerated followed by centrifugation at 5000 x *g* for 15 min at 4°C. The supernatant was recovered and passed through 0.45 µm and 0.22 µm filters (Millipore) and stored at -80°C.

### HrpB2 secretion by *Xac*

*Xac* 306 cells were cultivated at 30°C in MM medium (pH 5.4) plus 100 µg/ml carbenicillin containing sweet orange leaf extract (extract derived from 1 g of leaf tissue per litre of MM medium). *Xac* cultures (50 mL) were grown for 24 h to an *OD*_600_ = 0.3 after which cells were collected by centrifugation and resuspended in 3 ml of urea-SB: 8 M urea, 10% glycerol, 52 mM Tris-HCl (pH 6.8), 2% SDS, 0.1% bromphenol blue, 140 mM 2-mercaptoethanol. The extracellular (secreted) fraction from a 50 ml culture was passed through a low protein-binding filter 0.45 µm (Millipore). Proteins in the filtrates were precipitated by adding 10% trichloroacetic acid and freezing at −20°C for 12 h followed by centrifugation at 12000 x *g* (4°C). The precipitate was washed twice with cold acetone and resuspended in 50 µl urea-SB. Note that the above procedure produces a secreted fraction that is derived from 60 times as many bacterial cells per unit volume as the cellular fraction. Equal volumes of cellular and secreted protein fractions were separated by SDS-PAGE (18% acrylamide) and transferred onto the nitrocellulose membrane. HrpB2 was detected by Western blot using anti-HrpB2_XAC_ antibodies (above).
